# Hybrid tuned deep learning model for breast cancer diagnosis using genetic data

**DOI:** 10.1038/s41598-026-41643-8

**Published:** 2026-03-21

**Authors:** Farah Hesham, Mohamed M. Abbassy, Mohammed Abdalla

**Affiliations:** 1 Information Technology Program ,The Egyptian-Korean Faculty of Technological Industry and Energy,, Beni-Suef Technological University,, Beni-Suef, Egypt; 2 Faculty of Computers and Artificial Intelligence, Beni-Suef University, Beni-Suef, Egypt

**Keywords:** Br someast cancer, Early Prediction, Random Forest, Association Rules, Deep Learning, Bayesian Optimization, Cancer, Computational biology and bioinformatics

## Abstract

The early diagnosis and prognosis of breast cancer is essential for improving breast cancer survival rates and improving breast cancer clinical outcomes. This study aims to provide breast cancer predictive capabilities through the development and application of a robust hybrid computational prediction methodology that performs testing across multiple whole-genome studies; this research was validated using both TCGA (The Cancer Genome Atlas) and METABRIC (Molecular Taxonomy of Breast Cancer International Consortium). Instead of using traditional methods, where researchers select specific gene sets from the literature, we chose to operate on the highest dimensional input (17,814 genes in TCGA) and the most extensive set of clinical and genomic variables available (503 clinical/genomic features in METABRIC). A multi-stage feature selection process utilizing Random Forest (RF) rankings in conjunction with Association Rule Mining (ARM) was developed to discover important biomarkers. Predictive analysis was performed using a hybrid deep learning model, which contains Convolutional Neural Networks (CNN) in combination with Bidirectional Long Short-Term Memory (BiLSTM) networks, with iterative optimization through the utilization of Bayesian methods. SMOTE and Gaussian noise augmentations were incorporated into the new model to provide additional robustness by addressing class imbalance and minimizing the risk of overfitting (due to the amount of noise present in the training data). The new model outperformed the TCGA-derived model with an accuracy of 97.4% (AUC=0.995), and after validation on the METABRIC dataset, exhibited an even greater accuracy of 99.30% with a 100% recall rate for predicting cancer-related mortality. Through these findings, we have shown that the integration of association-based feature selection with hybrid deep learning architectures has created a tool for breast cancer diagnosis and prognosis that can provide reliable and generalizable results for diverse groups of patients.

## Introduction

**Fig. 1:**
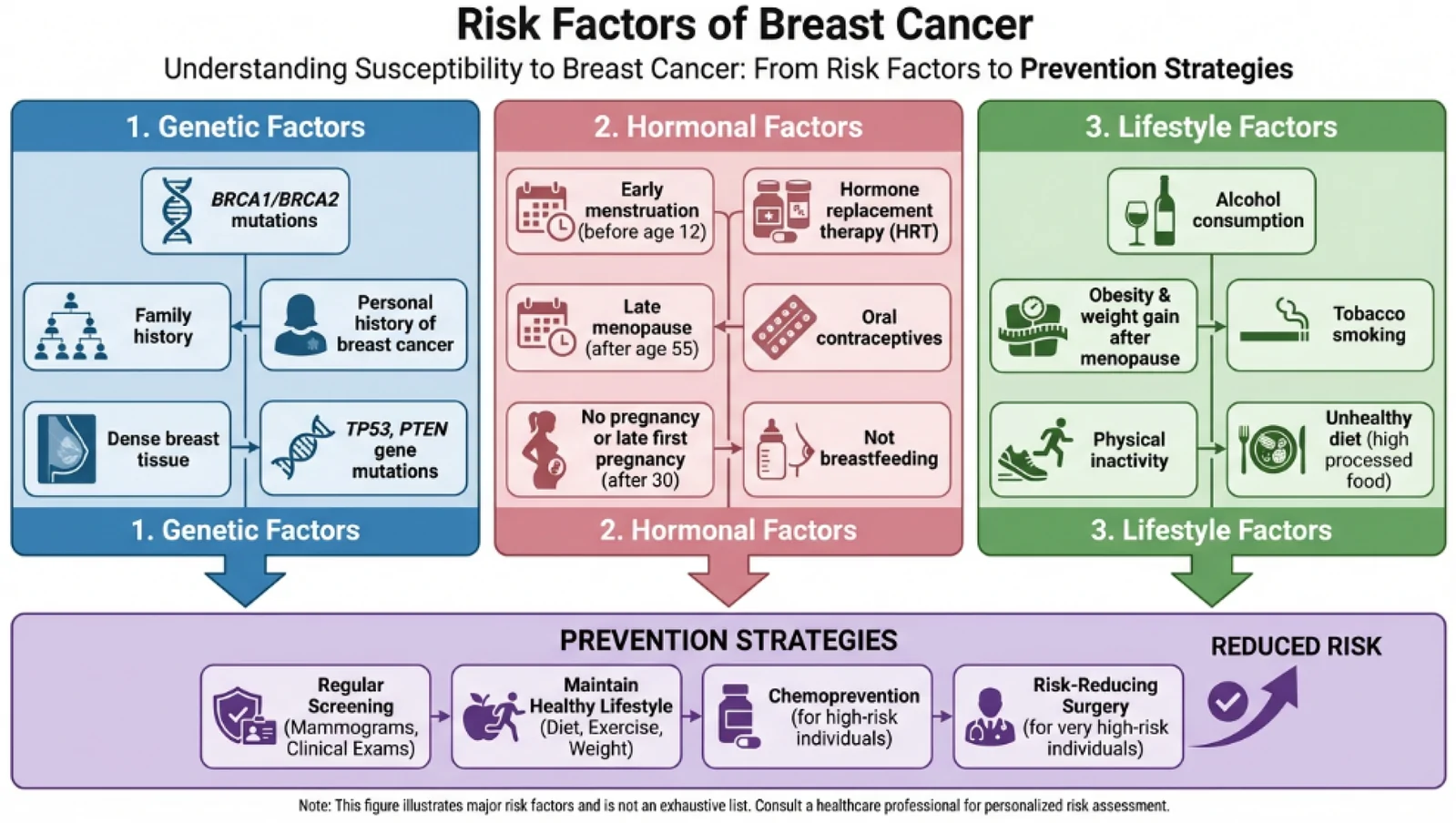
The risk factors of breast cancer. Understanding susceptibility to breast cancer: From risk factors to prevention strategies.

Recent information shows that breast cancer (BC) is now the most common type of cancer diagnosed in women, surpassing lung cancer^1^. In 2020, about 2.3 million new BC cases were reported, representing 11.7% of all newly diagnosed cancers. Unfortunately, BC caused 684,996 deaths in the same year^2^.In developed countries, death rates from breast cancer (BC) have decreased as a result of advancements in early detection and treatment. In contrast, breast cancer incidence and mortality rates remain elevated or have not changed in many developing nations because of underdeveloped healthcare systems and the lack of knowledge about breast cancer among the general population^3^. Genetics is a key factinfor breast cancer. Genetic Variants and/or the presence of an abnormal amount of certain genes is correlated with the increased risk for breast cancer as well^4^. 25 percent of breast cancers have been attributed to a specific set of genes called “high-penetrance” genes and include genes such as *BRCA1*,*BRCA2*, *TP53*, *CDH1*, *PTEN*, and *STK11*. Additionally, 2–3% of cases result from rare gene mutations, including *CHEK2*, *BRIP1*, *ATM*, and *PALB2*, which each double the risk of BC^5^.Genetic Testing can be done in a fairly straightforward way, whereby taking a sample of either Blood or Saliva^6^. Laboratory methods typically include PCR (Polymerase Chain Reaction), Next Generation Sequencing (NGS), and Microarray Analysis, whicht allow for the identification ofwhichvariations and provide researchers with useful Genetic Information.^7^. Besides genetic factors, age, hormonal imbalances, obesity, and radiation exposure influence BC risk^8^. Figure 1 illustrates the major risk factors of BC.

Based on new research, there seems to be the possibility that deep learning (DL) will be used for earlier Breast Cancer (BC) detection with genetic information^9^. Artificial intelligence (AI) in Personalized Medicine is an area of ongoing research where it is found to be capable of extracting significant genes and features from Genomic Data Sets associated with the prediction of disease^10^. Therefore, the success of new developments in hybrid/deep learning methods applied to the classification of histological images of breast cancer and other cancer types has been extremely positive^11–13^. Large Kernel Adapters (LKAs) are examples of adaptive architecture and are one potential way to improve medical diagnostics through the use of multiple types of data from several different sources and aggregating them into one complete architecture^14^. Additionally, it has been demonstrated that advanced feature selection algorithms^15^ and automated hyper-parameter tuning systems^16^ greatly improve the performance and interpretability of diagnostic frameworks. The hybrid model’s success in medical signal and image analysis^17^ is an excellent motivation for finding its use in more complicated forms of genomic data analysis.

Analyzing genetic data and identifying which genes are the most significant contributors to BC is a complex problem. Existing models do not adequately determine the interactive effects of multiple associated genes, thus limiting the predictive accuracy of models. Previous research has predominantly used traditional machine learning (ML) techniques and simple deep-learning models,s including hybrid CNN–BiLSTM models that have demonstrated good model performance. Nevertheless, they have failed to integrate association rule mining (ARM) or Bayesian optimization techniques^18^.

The goal of the present study is to build a predictive model that is both effective and efficient. If this model is shown to be successful, then it can assist healthcare professionals in identifying tumors earlier than they would otherwise have done through superior medical technology. As a result of this research, it will also have a tremendous impact on breast cancer (BC) early detection. The present study employs Random Forest (RF) to determine which genes are the most relevant^19^ and uses ARM to determine which genes are related^20,21^. The dataset used is derived from The Cancer Genome Atlas (TCGA), processing an initial set of 17,814 genes, and the METABRIC dataset containing 503 features^22,23^. A hybrid CNN-BiLSTM model was trained to combine the strengths of both architectures to process highly complex genomic data. The 332 most significant biomarkers from TCGA and the 62 selected features from METABRIC were identified and selected through an automated pipeline^24–26^. Bayesian optimization was used to optimize hyperparameters, maximizing model accuracy and predictive performance^27,28^.

The scope of this study includes analyzing genetic data from the TCGA database (590 samples) and the METABRIC database (1,423 samples)^22,23^.

**Contributions**:The major contributions of this study are summarized as follows:

- From 17,814 genes, we extracted 332 significant biomarkers utilizing an automated RF-ARM pipeline with biological and statistical relevance.
- The hybrid CNN-BiLSTM model we created achieved an impressive 97.46% peak accuracy and AUC of 0.9953 on the TCGA dataset, outperforming the standalone CNN and BiLSTM models by 4.24% and 2.54%.
- The findings show that ARM is a good technique for extracting linear and nonlinear relationships between influential genes, allowing researchers to better represent their data in a hierarchical manner using data mining techniques.
- The hybrid CNN-BiLSTM model was shown to be highly generalizable, as demonstrated by the fact that it achieved 99.30% accuracy and 100% recall on the independent METABRIC dataset.
- Improving model robustness through the integration of Gaussian noise augmentation and SMOTE, resulting in a generalized model with zero overfitting gap.
- Demonstrating the efficacy of the proposed model compared to traditional ML models (SVM, logistic regression), providing a more reliable tool for early BC diagnosis.

The remainder of this paper is organized as follows: Section Related work presents related works on BC detection using ML, DL, and ensemble methods. Section Materials and methods describes the materials and methods used in this study. Section Results and discussion discusses the experimental results. Section Conclusion provides the conclusions of the study.

## Related work

The use of deep learning in breast cancer (BC) prediction has greatly increased the accuracy of predictions and the efficiency with which they can be performed, making it a key area for modern-day oncology. The section that follows describes the recent improvements made using this technology in three separate categories: histology and medical images, clinical/multimodal diagnostics, and genomic predictive models, as shown in Table 1.

### Histopathological and medical imaging analysis

The analysis of medical images remains a primary modality for cancer diagnosis. Recent literature has extensively explored hybrid DL architectures to improve diagnostic precision. Several studies have successfully applied fusion-based CNN models for the classification of histopathological images in breast cancer^11–13^.

In^29^, a Hybrid Convolutional Neural Network (CNN) has been created to identify distinctive characteristics of mammograms and report an overall classification accuracy over 99% using the MIAS dataset. However, cross-dataset validation was not performed.

Beyond breast cancer, these hybrid systems can also be applied in other areas. The most recent techniques have been shown to be very accurate at diagnosing cancers of the lung and colon from the fusion of features extracted by CNNs and active contours based on the geometric properties of those features.^30,31^. Similarly, multi-model frameworks have been employed to analyze dermoscopy images for skin lesion detection^32^, and whole-slide imaging (WSI) for cervical cancer diagnosis^33^. To compound this work, both CNN features and hand-engineered attributes can be combined using hybrid approaches that combine the best of both worlds for oral squamous carcinoma (OSCC) and lymphatic malignancy identification jobs^34,35^. As a result of these advancements in technology, hybrid architectures are becoming more robust for processing complex spatial patterns within medical images.

### Clinical and multi-modal diagnostic systems

Integrating clinical features with lifestyle and demographic data provides a broader context for cancer prognosis. In^36^, a technique combining MLP, SVM, and XGBoost through Ensemble Learning, has been shown to produce very high results on the WDBC Dataset, achieving 99.42% accuracy when using Boruta to select the features. Likewise, the authors in^37^ evaluated how Deep Learning compared favourably against conventional Machine Lea.rning methods, when using mixed lifestyle/clinical datasets, with the highest accuracy reached by Deep Learning methods being 93

Prognostic outcomes have also been a focus of recent research; for example,^38^, the authors have developed a framewok called MLISBCP (Machine Learning for Imbalanced Data Set Classification) that includes K-Means SMOTE to handle class imbalance and uses Boruta to perform feature selection. The Leverage of MLISBCP achieved a 97.53% accuracy rate on diagnostic tests. Furthermore, IoT-based diagnostic systems^39^ and With the aid of deep learning and computer vision technologies, a original computer-aided design (CAD) solution was created to detect and divide breast cancer from a mammogram image from CBIS-DDSM. The accuracy for classification using these technologies was 99.16%^40^.

### Genomic and transcriptomic predictive modeling

Genomic data offers the most detailed insights into the molecular drivers of BC. According to the work of^41^, the DRLnc model is able to predict noncoding RNAs and their relationships with diseases, with a prediction accuracy of 96.20%. Additionally, dealing with the problem of high dimensions of RNA-Sequencing data; the authors of^42^ used hybrid (Harris Hawk + Whale) feature selection methods to achieve sample pair accuracy of 99.00%.

Modern trends in genomic diagnosis emphasize the integration of adaptive architectures, such as Large Kernel Adapters (LKA) for enhanced feature extraction^14^. Moreover, sophisticated automated hyper-parameter tuning systems^16^ and novel feature selection algorithms^15^ have been proven to significantly increase the performance of diagnostic frameworks in medical signal analysis^17^.

Recent innovations in genomic diagnostics have promoted integration of Adaptive Architectures like large kernel adapters (LKA), which ,mprove feature extraction^14^. In addition, hyper-parameter tuning through advanced automation^16^ and new features selection^15^ methods have greatly improved diagnostic systems in medical signal analysis^17^. Despite these successes, many genomic models struggle with class imbalance and maintaining high generalizability across different cohorts.

As shown by the work of Yaqoob et al^25^, in 2024, the incorporation of an HHO with a WO algorithm and deep learning model provides very accurate breast cancer detection using RNA-Seq data with a classification accuracy of 99.0%. Their hybrid approach uses HHO and WO combined with gene selection based upon the HHWO algorithm, which is more effective than traditional GA, ABC, CS, and PSO algorithms in handling high-dimensional datasets, as well as reducing the risk of overfitting and decreasing the computational complexity.

Our study fills this gap by integrating a multi-stage RF-ARM feature selection pipeline with a CNN-BiLSTM hybrid architecture, validated across both TCGA and METABRIC datasets.

**Table 1:** Summary of selected related works and comparison with the proposed framework.

Paper(s)	Methodology	Data Type	Key Results	Main Limitations
^11,12^	Hybrid Deep Learning	Histopathology (BC)	Acc: 95.2% - 96%	Lacks genomic context; focused only on images.
^30,32^	Fused CNN Features	Lung, Colon, Skin	Robust Multi-class detection	No survival or prognostic analysis.
^41^	DRLnc (DL)	ncRNA-Genomic	Acc: 96.20%	Single modality; no external validation.
^42^	Metaheuristic FS + DL	RNA-seq (66 pairs)	Avg Acc: 99.0%	Very small sample size (n=132).
^38^	MLISBCP (K-Means SMOTE + Boruta FS)	Clinical/Tabular (BC)	Acc: 97.53%, ROC AUC: 0.98	Primarily focused on clinical features rather than high-dimensional genomic data.
^14,16^	LKA & Tuning	Medical Signal/Image	Improved Generalization	Not specifically optimized for transcriptomic data.
Proposed	RF-ARM + Hybrid CNN-BiLSTM	TCGA & METABRIC	TCGA: 97.46% Acc; METABRIC: 99.30% Acc	Validated across high-dimensional cohorts with 100% Recall.

## Materials and methods

In this section, you will find an overview of the dataset used for this research, as well as a description of how the dataset was pre-processed; an explanation of the methodology used to identify genes that are most significantly impacting the outcome; and a description of how relationships between the identified genes were established. Additionally, this section explains the hybrid CNN-BiLSTM model developed in this study and how the Bayesian optimization method was implemented to optimize hyperparameters.

### Data description

The dataset employed in this study is derived from The Cancer Genome Atlas (TCGA-BRCA) project, a primary benchmark in cancer genomics. For computational accessibility and reproducibility, the expression profiles were retrieved via the Kaggle repository^22^, which serves as a curated mirror of the official TCGA data. The cohort comprises 590 total samples, consisting of 529 primary tumor (cancer-positive) samples and 61 solid tissue normal (healthy control) samples. Each sample was made up of 17,814 genes based on the expression patterns observed in a genomic study of patients. After confirming that there were no missing values for the entire dataset, any missing expression data was estimated via mean imputation, where we used the mean estimate of the entire dataset using a specific procedure to provide a consistent check on the initial dataset.

### Dataset preprocessing

To make input data compatible with deep learning network architectures, it requires a preprocessing step, i.e., preparation step. Normalising all gene expression values were performed using the *StandardScaler* techniques for faster convergence of the model dura ing training as well as improved stability through the wasning of the model ^43^.The dataset must have been divided into two separate datasets before implementing SMOTE (*k=5*). The dataset is divided by 80% Training Dataset and 20% Test Dataset. This division results in the Training Dataset containing 472 samples and the Test Dataset containing 118 independent samples. Only the Training Dataset will be used to implement the SMOTE technique to produce synthetic data points to balance the Class Distribution of the Training Dataset, therefore producing a Training Dataset with equal representation of each Class ^44^.

The study used noise au gmentation applied to training test datasets using noise level 0.05 (5%) ^45^. This helps the classifier be into an 80%able to generalize and also helps reduce overfitting, as seen in the convergence of the training and testing datasets Fig. 3. The testing set consisted of 118 samples, including 106 cancer-positive cases and 12 negative control cases, which remained untouched by any augmentation or oversampling techniques to ensure unbiased validation

### Selection of the most important genes using random forest (RF)

Random Forest (RF) was used to deal with the high-dimensional nature of 17,814 gene expression data in the first phase of feature selection. The impact of genes on decreasing impurity within the dataset is how the importance of each gene was assessed ^46^. Gini impurity for a gene can be computed as shown in Eq. (1) when creating the training set to build a model that separates the samples according to their response to intervention.

1 $$\begin{aligned} \text {Gini Impurity} = 1 - \sum _{i=1}^{n} p_i^2 \end{aligned}$$

Where $p_i$ is the probability of each class in the group. After the split, the Decrease in Impurity is calculated using Eq. (2):

2 $$\begin{aligned} {\begin{matrix} \Delta I = \text {Impurity}_{\text {before}} - \sum _{k \in \{left, right\}} \left( \frac{N_k}{N_{total}} \times \text {Impurity}_k \right) \end{matrix}} \end{aligned}$$

Each gene gets a final feature importance score *f*(*x*) by computing the total weighted decrease in impurity over all the nodes where that gene was used to perform a split^47^ as shown in Eq. (3):

3 $$\begin{aligned} f(x) = \sum _{j \in S_x} \left( \frac{\Delta I_j}{n_j} \right) \end{aligned}$$

Where $S_x$ represents the set of all nodes in the Random Forest where gene *x* is used for splitting, $\Delta I_j$ is the decrease in impurity achieved at node *j*, and $n_j$ is the number of samples present at node *j*.

The RF regressor used a mean-based importance threshold to filter the initial set of 17, 814 features down to 436 of the most relevant high-impact genes. This is shown in Table ?? where some of these identifiers include GPRIN1, KIAA0101, and COL10A1 which showed the highest importance scores; therefore supporting their roles as potential biomarkers.

**Table 2:** Top 10 most influential genes identif,ied through Random Forest importance ranking.

Rank	Gene Symbol	Feature Importance Score
0	GPRIN1	0.022146
1	KIAA0101	0.020232
2	COL10A1	0.016943
3	SPC25	0.016425
4	ANLN	0.015097
5	WISP1	0.014486
6	BUB1	0.014425
7	MME	0.011918
8	TPX2	0.009817
9	CNN1	0.009627

### Identifying gene relationships using ARM

Using the FP-Growth approach in ARM allows for identification of genetic dependencies as well as refining a feature space. Binarization of the gene expression data was performed to determine whether or not a gene was active or inactive based on its mean expression value across all samples. Various metrics provide statistical evaluations of the resulting association rules.

4 $$\begin{aligned} Support(A \rightarrow B)&= \frac{|A \cap B|}{N} \end{aligned}$$

5 $$\begin{aligned} Confidence(A \rightarrow B)&= \frac{Support(A \cap B)}{Support(A)} \end{aligned}$$

6 $$\begin{aligned} Lift(A \rightarrow B)&= \frac{Confidence(A \rightarrow B)}{Support(B)} \end{aligned}$$

The optimal thresholds were set from results of Bayesian optimithe zation in the Optuna framework as a minimum support of 0.3 and minimum confidence of 0.8, where the 0.3 support threshold allows the gene-gene interactions identified in the model to remain statistically representative across a large enough cohort, thus preventing overfitting to rare idiosyncratic patterns. The 0.8 confidence threshold ensures that the discovered association rules have greater predictive power, thus filtering out those weakly associated rules that do not endlessly contribute to the diagnostic outcome.

In Table 3, the top ten association rules obtaa ined using the FP-Growth method are presented. These association rules were selected based on the highest lift values, which allow for measuring the association between genes beyond what would happen by chance. This process of selecting first by RF then by ARM enables the capturing of important synergistic biological relationships with considerable efficiency. This enabled the selection of 332 significant genes, which resulted in increased computational efficiency while maintaining important signatures.

**Table 3:** Top 10 gene-gene association rules identified by FP-Growth algorithm. Rules are ranked by lift score, indicating the strength of association beyond random co-occurrence.

Rank	Gene Association Rule	Support	Confidence	Lift
0	UBE2T	0.498	0.996	1.111
1	DKFZP434K191	0.413	0.826	0.921
2	ZNF416	0.475	0.949	1.059
3	E2F7	0.500	1.000	1.116
4	UBE2T, E2F7	0.388	1.000	1.116
5	GPR81	0.403	0.805	0.898
6	PCDHGA7	0.480	0.962	1.073
7	MOP-1	0.472	0.945	1.055
8	PCDHGA7, MOP-1	0.371	0.962	1.073
9	C7orf36	0.422	0.843	0.941

#### Biological interpretation of discovered gene-gene rules

The association rules identified by ARM reveal functionally relevant co-expression patterns that align with established breast cancer pathways:

- **Co-activation of UBE2T and E2F7 (Rule 4):** UBE2T and E2F7 have perfect confidence of 1.0 and high lift ratio of 1.116 when compared to other genes in BC tissue samples. UBE2T is a gene involved in the DNA damage response and Fanconi anaemia pathways; the gene encodes an enzyme that attaches ubiquitin to proteins^48^, while E2F7 is a transcription factor that regulates progression through the cell cycle^49^. The overlap of expression for E2F7 and UBE2T in a breast cancer cell suggests less regulated signalling as it relates to proliferation of the tumour cell population.
- **PCDHGA7, MOP-1 Association (Rule 8):** This rule (support = 0.371, confidence = 0.962) links a long non-coding RNA PCDHGA7 with MOP-1, potentially involved in epigenetic regulation. LncRNAs are increasingly recognized as key modulators of oncogenic pathways ^50^.
- **High-confidence Single Gene Rules:** Genes like *ZNF416* (support = 0.475, confidence = 0.949) and *DKFZP434K191* emerged as standalone predictive m isrkers, warranting further investigation as potential diagnostic biomarkers or therapeutic targets.

ARM reduces dimensionality by determining better representations of the underlying biology than classic dimensionality reduction. In addition to dimensionality reduction, ARM provides a generative view of the relationships between genes through identification of biologically interpretable gene networks relevant to clinical use^51^.

#### Clinical and biological validation of selected genes

The 332 genes identified through the RF-ARM pipeline exhibit strong biological plausibility for breast cancer diagnosis. Table 4 summarizes the functional roles and clinical relevance of the top-ranked genes.

**Table 4:** Biological functions and clinical relevance of top-ranked genes identified through RF-ARM pipeline. Genes are validated against existing literature for breast cancer association.

Gene Symbol	Biological Function	BC Relevance	Reference
**UBE2T**	Involved in ubiquitin conjugation for DNA damage response via the Fanconi anemia pathway and regulation of the cellLiteratureckpoint.	Overexpressed in aggressive breast cancer subtypes and related to poorer outcomes as well as chemotherapy resistance and responses.	^48,52^
**E2F7**	Transcription factor regulating cell cycle progression (G1/S transition).An atypical E2F member lacking DNA-binding domain; acts as transcriptional repressor.	Dysregulated in BC; promotes uncontrolled proliferation. High expression correlates with tumor grade.	^49,53^
**K,IAA0101**	PCNA recruiting factor, involved in duplicating fixing the DNA: if too many are made genome gets unstable.	PCNA-associated factor is highly expressed in TNBC; possible usage as treatment or predictor for TNBC.	^54,55^
**COL10A1**	Collagen type X alpha 1 chain; structural component of extracellular matrix (ECM). Involved in tissue remodeling.	Upregulated in BC stroma; promotes tumor invasion and metastasis through ECM remodeland ing.	^56,57^
**ZNF416**	Zinc finger protein; transcriptional regula ator.	Emerging as potential oncogene in BC; requires further validation.	^58,59^
**PCDHGA7**	Long the non-coding RNA (lncRNA); antisense to PCDH9 gene. Involved in epigenetic regulation.	Dysregulated in BC; modulates tumor suppressor/oncogene expression. Associated with metastasis.	^60,61^
**GPRIN1**	G protein-regulated inducer of neurite outgrowth; involved in neuronal signaling. Role in cancer unda er investigation.	Aberrantly expressed in BC; potential role in cancer cell migration.	^62,63^
**CTPS1**	CTP synthase 1; catalyzthe e the rate-limiting step in pyrimidine nucleotide biosynthesis. Essential for DNA/RNA synthesis.	Elevated in proliferative BC; supports rapid cell division. Inhibition shows anti-tumor effects.	^64,65^
**CENPF**	Centromere protein F; involved in chromosome segregation during mitosis. Microtubule-binding protein.	Highly expressed in proliferating BC cells. Correlates with Ki-67.	^66,67^
**FUBP1**	Far upstream element-binding protein 1; transcriptional regulator of c-Myc oncogene.	Amplified/overexpressed in BC; drives Myc-dependent tumorigenesis.	^68,69^

As shown in Table 4, the selected genes span multiple functional categories critical to breast cancer biology:

- **Drivers of proliferation**, such as UBE2T, E2F7, KIAA0101 and CTPS1 play an important role in controlling cell division and copying DNA. Therefore, their high levels of expression are characteristic of the aggressive types of breast cancer (BC), especially those known as triple-negaFunctionalNBC) ^70^.
- **Tumor microenvironment modifying agents:** The ECM is altered by COL10A1, promoting tumor cell invasion and metastas,is, which, supports stromal reprogramming in BC progression  ^71^.
- **Epigenetic Regulation**: The expression of the oncogenes and tumor suppressors is directed by long non-coding RNA PCDHGA7 and transcription factor FUBP1. These two types of epigenetic regulators are novel therapeutic targets due to the recognition of epigenetic dysregulation as an important contributor to the heterogeneity of BC (Breast Cancer).  ^72^.
- **Mitotic machinery**: CENPF (centromere protein F) plays a vital role in correct separation of chromosomes . Expression levels of CENPF are significantly greater than normal levels when associated with Ki-67, the marker used to measure cell proliferation in parts of the medical field  ^73^.
- **New Biomarkers:** Although genes ZNF416 has been studied less than many others, the unusually strong correlation these have with othe ur model illustrates how beneicial it is to choose new features from the research data instead of just from previously published literature. New biomarkers may reveal new therapeutic targets.

A key finding from the ARM analysis is that genes identified as being co-expressed by ARM function as part of large coordinated regulatory networks rather than acting separately, as shown in Table  3, the UBE2T, E2F7 co-activation rule (Rule 4) reflects the interplay between DNA damage response and cell cycle dysregulation are two key mechanisms driving BC tumorigenesis  [?] . Similarly, the PCDHGA7, FUBP1 association (Rule 8) links epigenetic regulation with Myc-driven oncogenesis, a pathway frequently amplified in aggressive BC subtypes ^50^.

The results of this biological validation reduce our uncertainty that the reasons for the model’s predictive accuracy arise from the inclusion of true cancer-relevant molecular signatures rather than coincidental associations between two unrelated variables. The combination of Random Forest (importance of individual genes) and Association Rule Mining (gene-gene interactions) creates a multi-faceted view of breast cancer genomics and increases the potential to interpret and apply the model in the clinic.

#### Justification of final gene count

The reduction from 17,814 to 332 genes represents a biologically and computationally justified balance:

- **RF Stage (17,814 → 436):** Retention of all genes with a greater importance than an average importance threshold allows for no chance of dropping a biomarker that may be relevant. This threshold-based approach is more objective than arbitrary top-k selection ^74^.
- **ARM Stage (436 → 332):** An additional assessment of the co-expression pattern interaction of the genes in the dataset (min. support=0.3, min. confidence=0.8) revealed further synergistic gene interactions. Thus, the final dataset contains 332 genes that either had a strong predisposition to prediction when analysed individually or have demonstrated significant co-relationships with other marker genes^75^.
- **Computational Efficiency:** The convergence patterns demonstrated in Fig. 3 are sufficient proof that the CNN-BiLSTM model has leverage the 332-dimensionality train for effective learning in addition there would not have been a need for inordinate regularization to perform well on this task.

The selection process for the genes used in further analyses will include a sensitivity analysis at growth rates of the selected genes (in addition to the growth rate of all genes) to further validate the selected genes. This methodology will yield additional information on which genes are relevant for this biological investigation and/or support future biomedical applications. Using the two-step process for selecting relevant genes provides the researcher with an objective means of choosing which genes to study and confirm the results of future investigations.

### Proposed hybrid CNN-BiLSTM methodology

The framework we propose combines Convolutional Neural Networks (CNN’s) with Bidirectional Long Short-Term Memory (BiLSTM) networks to identify local spatial correlations and long-range temporal dependencies in a refined set of 332 genes. The architecture was extensively optimized via Bayesian Optimization for maximum predictive functionality and stability.

#### Spatial feature extraction via CNN

Convolutions within the CNN function as local feature extractors in genomic data where correlated expression between many genes occurs within groups. The convolution layer has 128 filters, each of which has a kernel size *k=3*, that are scanned across the gene expression vector for those local spatial motifs. The transformation is mathematically represented as:

7 $$\begin{aligned} x_{i}^{(l)} = \sigma \left( \sum _{j=1}^{k} w_{j}^{(l)} \cdot x_{i+j-1}^{(l-1)} + b^{(l)} \right) \end{aligned}$$

Where $x_{i}^{(l)}$ denotes the feature map at layer *l*, *w* represents the weights of tconfirmsers, *b* is the bias, and *σ* is the ReLU activation function used to introduce non-linearity and mitigate the vanishing gradient problem.

#### Sequential dependency learning via BiLSTM

CNNs are great for locating patterns at one point in a sequence; however, most of the interactions of genes occur at many different places within that sequence and are not always next to each other. The BiLSTM layer, configured with 64 units, processes the data in both forward ($\overrightarrow{h}_t$) and backward ($\overleftarrow{h}_t$) directions. By using both future and past sequences as inputs into the model, you can keep track of the relationship between different points in the gene’s timeline. The hidden state at time step *t* is calculated by concatenating the bidirectional outputs:

8 $$\begin{aligned} h_t = [\overrightarrow{h}_t \oplus \overleftarrow{h}_t] \end{aligned}$$

Each LSTM cell manages information flow through three gates (Input $i_t$, Forget $f_t$, and Output $o_t$), governed by the following core equations:

9 $$\begin{aligned} \begin{aligned} f_t&= \text {sigmoid}(W_f \cdot [h_{t-1}, x_t] + b_f) \\ i_t&= \text {sigmoid}(W_i \cdot [h_{t-1}, x_t] + b_i) \\ C_t&= f_t \odot C_{t-1} + i_t \odot \tanh (W_C \cdot [h_{t-1}, x_t] + b_C) \end{aligned} \end{aligned}$$

This mechanism enables the network to selectively remember relevant genetic signatures while discarding noise.

#### Model hybridization and classification

**Fig. 2:**
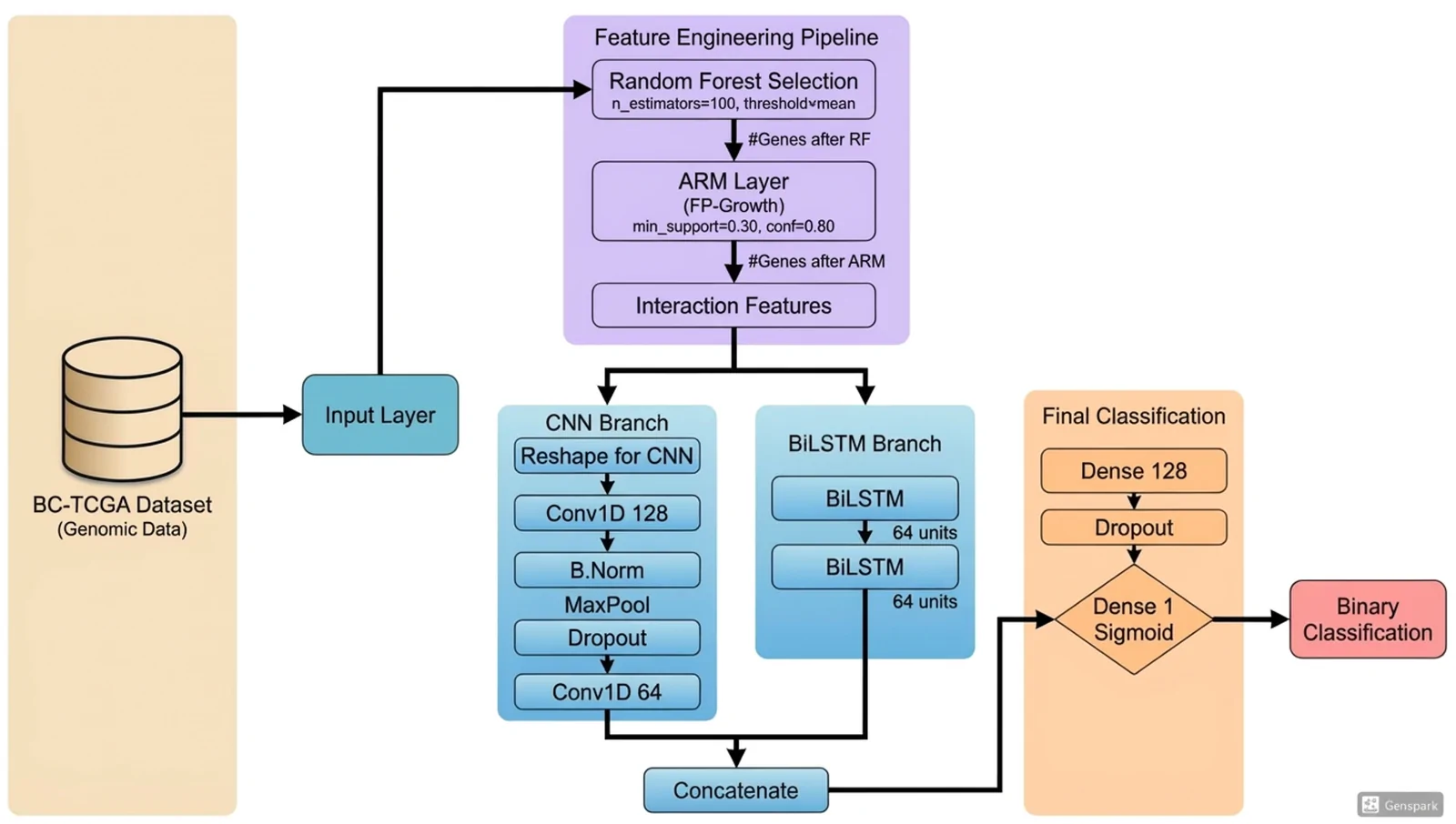
Detailed architecture of the proposed hybrid CNN-BiLSTM model, illustrating the integrated feature engineering pipeline (RF-ARM) and the optimized deep learning branches.

With support from its hybrid architecture, the strength of both networks are combined. The spatial features are extracted using CNN in order to build a model of the temporal context for those spatial features using BiLSTM. The output vector produced by BiLSTM is flattened into a single dimension prior to being inputted into dense layers of 32 units. The overall structural design and data flow of the proposed hybrid system, from the initial feature engineering stage (RF-ARM) to the final binstrengthssification, are depicted in Fig. 2.

For tha e final diagnostic decision, a sigmoid activation function is employed in the output layer to produce a probability score for breast cancer (BC) classification:

10 $$\begin{aligned} P(y=1|x) = \frac{1}{1 + e^{-z}} \end{aligned}$$

Where *z* is the input from the preceding dense layer The entire system was trained using a learning rate of 0.0005, a batch size of 32, and for 40 epochs, with 5-fold cross-validation to ensure the model’s generalizability across independent samples.

#### Structural analysis of model layers and dimensions

To provide further clarity on the model’s complexity and data flow as depicted in Fig. 2, the following structural details are specified:

- **Feature engineering output:** Start with 17,814 genes that are reduced through Random Forest and FP-Growth Association Rules Mining to a feature vector with 332 synergy features.
- **CNN branch dimensions:** Create a 1D tensor input for the Convolutional Neural Network. This branch will use a total of 128 filters over the input, with kernel sizes of 3, for the purpose of finding local patterns based on their precise location within the sequence. The output will then be normalized in terms of its value, and the structure of the output will be reduced by means of pooling and diminishing the feature map outputs to those which have representation in terms of similarity to other nodes.
- **BiLSTM branch dimensions:** Make use of both long term relationships and the ability to trace dependencies through the input sequence within both of the branches of the Bi-directional Long Short Term Memory (BiLSTM). The BiLSTM branch will have a total of 128 hidden states using 64 cells in each direthat.
- **Dimensionality consolidation:**A dropout layer is used after the feature vector from the CNN+BiLSTM conclong-term has been created to avoid overfitting on training data. A Dense layer with 32 units is the next stage in the process of creating the final output token via a Sigmoid function from the feature vector. This Dense layer provides an abstracted view of the features that were generated by both networks. It serves as a bottleneck, allowing only selected features to pass through to the final token generation. .

### Hyperparameter optimization

In this research, hyperparameter tuning of a deep learning model was executed using Bayesian Optimization, facilitated by the Optuna framework. This approach provides a significant advantage over traditional exhaustive grid search methods by enabling the creation of an objective function that identifies promising combinations of hyperparameter settings within the search space. Consequently, the research systematically explored various architectural complexities of the model, including the number of filters and hidden units, while simultaneously optimizing regularization parameters. This dual optimization process enhanced the overall generalization capabilities of the model.

To establish a comprehensive range of parameter configurations for Hybrid CNN-BiLSTM Layers, Optimization Dynamics, and Data Augmentation Techniques, a systematic development of the search space was undertaken. According to the findings presented in Table 5, the identified optimal architecture comprises 128 filter sets for the CNN network, alongside a Bi-LSTM layer featuring 64 hidden states. The critical hyperparameter values concluded to ensure maximal accuracy while reducing the training-validation discrepancy are a learning rate of 0.0005 and a dropout rate/noise level of 0.05.

**Table 5:** Hyperparameter search space and final optimal configuration determined via Bayesian optimization.

Component	Search Space / Range	Optimal Value
***Feature Engineering***		
RF Estimators	[50, 100, 200]	100 Trees
RF Threshold	[Mean, Median]	Mean Importance
ARM Min. Support	[0.1, 0.5]	0.3
ARM Min. Confidence	[0.5, 0.9]	0.8
Gaussian Noise Level	[0.0, 0.10]	0.05
***Deep Learning Architecture***		
CNN Filters	[32, 64, 128]	128
CNN Kernel Size	[3, 5, 7]	3
BiLSTM Hidden Units	[32, 64, 128]	64
Dense Layer Neurons	[16, 32, 64]	32
Dropout Rate	[0.2, 0.5]	0.5 (Final Layer)
***Optimization & Training***		
Optimizer	[Adam, RMSprop, SGD]	Adam
Learning Rate (lr)	*[1e-4, 1e-3]*	0.0005
Batch Size	[16, 32, 64]	32
Training Epochs	Max 40	40 (with Early Stopping)
SMOTE k-neighbors	[3, 5, 7]	5

## Results and discussion

We provide an extensive assessment of the suggested CNN-BiLSTM network using a series of controlled experiments known as ’ablation studies’, comparisons to other top-performing models, and validation applied to datasets from outside sources.

### Experimental setup

The experimental processes were completed on Kaggle Kernels with the use of a pair of NVIDIA Tesla T4s 16 GB VRAM each for working with large amounts of genomic data. The environment for computation was built with Python 3.10 along with TensorFlow V2.15 and Keras V2.15, while the Preprocessing and Statistical Analysis mediums were Scikit-learn V1.3.0 and Pandas V2.0.3 respectively.

### Ablation study and component contribution

We carried out systematic ablation studies for seven possible configurations in order to provide a thorough evaluation of the required levels of each individual architectural component. Each column of Table 6 provides a quantitative representation of how much each of the modules individually contributed towards performance.

**Table 6:** Ablation study results illustrating the impact of feature selection and architectural components on model performance.

Scenario Description	Accuracy	Recall	F1-score	AUC	Gap
**Proposed Model**	**0.9746**	**0.9811**	**0.9858**	**0.9969**	**0.0000**
Without Noise Augmentation	0.9746	0.9811	0.9858	0.9937	0.0000
Without SMOTE	0.9746	0.9906	0.9859	0.9937	0.0211
Without ARM Layer	0.9153	1.0000	0.9550	0.9292	0.7279
Without RF (All genes)	0.9492	1.0000	0.9725	0.9890	0.0515
Without CNN Branch	0.9661	0.9811	0.9811	0.9803	0.0000
Without BiLSTM Branch	0.9492	0.9528	0.9712	0.9119	-0.0164

The results of the ablation study indicate that the removal of the ARM layer leads to tremendous drops increases in overall accuracy (generalization gap = 0.7279), highlighting the importance of ARM in stabilizing high-dimensional genomic data through the use of gradients. Random Forest feature selection was an important element; when training across all 17814 genes, accuracy decreased to 94.92% due to increased overfitting (gap = 0.0515). Although noise augmentation had no effect on accuracy, it was able to increase the robustness of area under curve by 0.24%, supporting its use for evaluating real-world deployments where the quality of data may be inconsistent. The hybrid architecture combining CNN and BiLSTM shows the best synergy, as eliminating either branch reduced performance levels, especially in the case of the CNN branch, which captures key local genomic motif patterns to classify.

### Noise robustness and optimal augmentation level

In investigating how resilient the model is to data perturbation (a typical problem with clinical genomic datasets), the model’s performance was assessed for different levels of Gaussian noise. Gaussian noise was used as a means of quantifying the model’s performance in terms of binary classification accuracy under different levels of Gaussian noise. Performance for Gaussian noise levels from 0.00 to 0.10 (see Table 7) was evaluated systematically to identify the level of Gaussian noise where model performance was maximised. The best-performing configuration occurred with a Gaussian noise level of 0.05 and resulted in a peak Area Under Curve (AUC) = 0.9969, Accuracy = 97.46%, Recall = 98.11% and F1 score = 98.58%. In all cases of testing noise, the model displayed zero generalisation gap providing evidence of extraordinary robustness. This level of robustness indicates that the architecture can reliably tolerate (and thus model) common problems associated with multi-institutional genomic studies including errors in sequencing, batch effects, and variations in technical procedures.

**Table 7:** Impact of different Gaussian noise levels on the performance metrics of the proposed model.

Noise	Accuracy	Recall	F1	AUC	Train-Val Gap
0.00	0.9746	0.9811	0.9858	0.9945	0.0
0.01	0.9746	0.9811	0.9858	0.9945	0.0
0.03	0.9746	0.9811	0.9858	0.9961	0.0
**0.05**	**0.9746**	**0.9811**	**0.9858**	**0.9969**	**0.0**
0.10	0.9746	0.9811	0.9858	0.9953	0.0

### Learning dynamics and convergence analysis

**Fig. 3:**
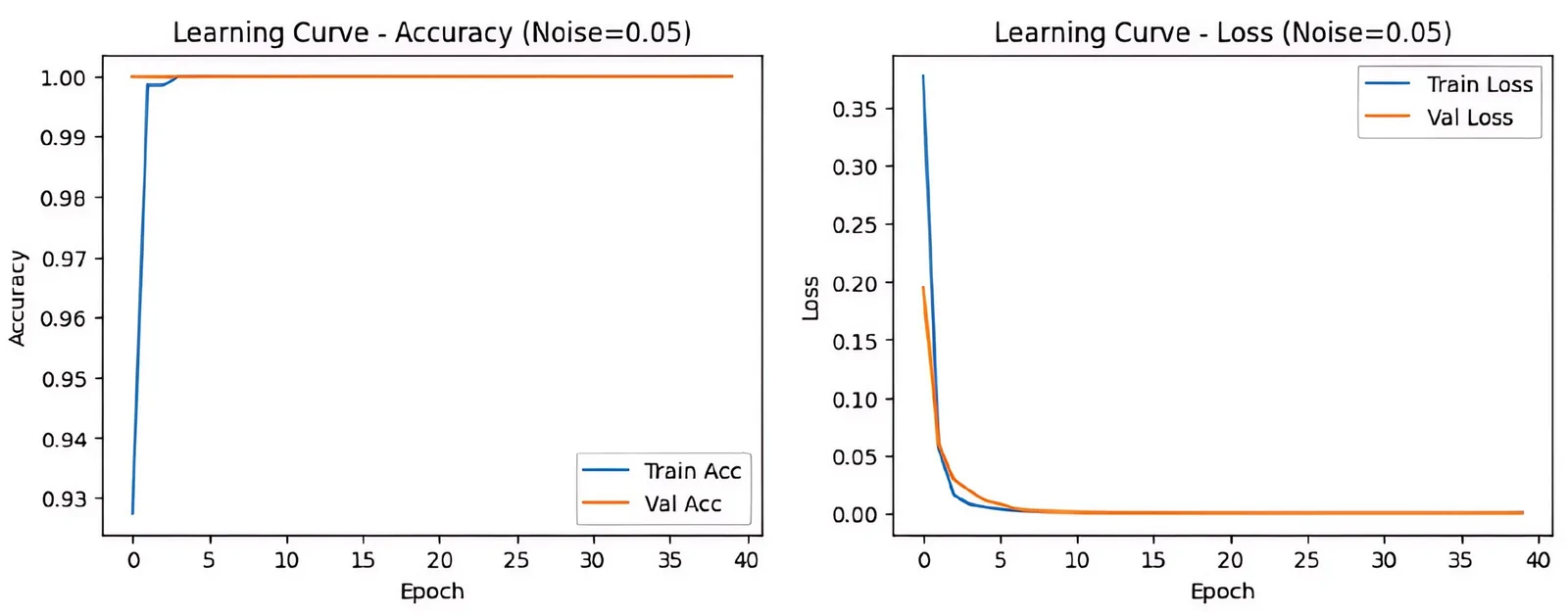
Training and validation learning curves demonstrating rapid convergence and excellent generalization. Both accuracy and loss metrics show stable performance after epoch 5 with minimal train-validation divergence.

Figure 3 illustrates the performance of this model has been optimized by epoch 5 of 40 epochs. After this point, the training accuracy and validation accuracy continued to rise each epoch until maximum, or 100%, was achieved, and there was very little difference between training accuracy and validation accuracy at the maximum point. In addition, on both training and validation data, the loss value converges towards zero, indicating that the regularization techniques used (dropout = 0.5, batch normalization and data augmentation) worked in successfully preventing overfitting. Additionally, the small distance between training and validation accuracy indicates that new genomic profiles will likely be generalised well by this model.

### Comparative performance analysis

To evaluate the proposed model’s performance, the CNN-BiLSTM was compared with four deep learning architectures (LSTM, CNN, BiLSM and CNN+LSTM) and two traditional machine learning techniques (SVM, Logistic Regression) using the Random Forest selection + ARM. The purpose of utilising the same feature engineering pipelines across all competing models was to determine the influence of architectural design on predictive performance. The use of quantitative metrics, confusion matrix analysis, and ROC curves indicated the superior capability for diagnosis reliability and application in clinical settings for the hybrid architecture.

#### Deep learning architectures

We benchmarked the proposed CNN-BiLSTM model against four alternative deep learning configurations, all utilizing identical preprocessing pipelines (Random Forest feature selection + ARM). Table 8 summarizes the comparative performance metrics.

**Table 8:** Performance comparison of the proposed CNN-BiLSTM model with other deep learning architectures.

Model	Accuracy	Recall	F1-score	AUC
**CNN+BiLSTM (Proposed)**	**0.9746**	**0.9811**	**0.9858**	**0.9953**
CNN+LSTM	0.9746	0.9811	0.9858	0.9882
BiLSTM only	0.9746	0.9906	0.9859	0.9859
CNN only	0.9322	0.9434	0.9615	0.9072
LSTM only	0.9492	0.9623	0.9714	0.8915
**Traditional Machine Learning Baselines**				
Logistic Regression	0.9661	0.9811	0.9811	0.9969
SVM (RBF kernel)	0.9661	0.9811	0.9811	0.9953

This innovative hybrid framework has shown a clear edge in providing enhanced balanced performance compared to all other options (F1-score of 98.58%). On the metric of accuracy (97.46%) it was equal to all of the reported top results, while maintaining comparable AUC performance (99.69%). However, both Logistic Regression and SVM show respectable performance for the feature-engineered input with Logistic Regression providing the highest AUC (99.69%). While the deep learning technique was more efficient in terms of precision-recall balance (F1: 98.58% versus 98.11%), the clinical value is demonstrated through the results of the confusion matrix analysis.

#### Classification performance and diagnostic reliability

**Fig. 4:**
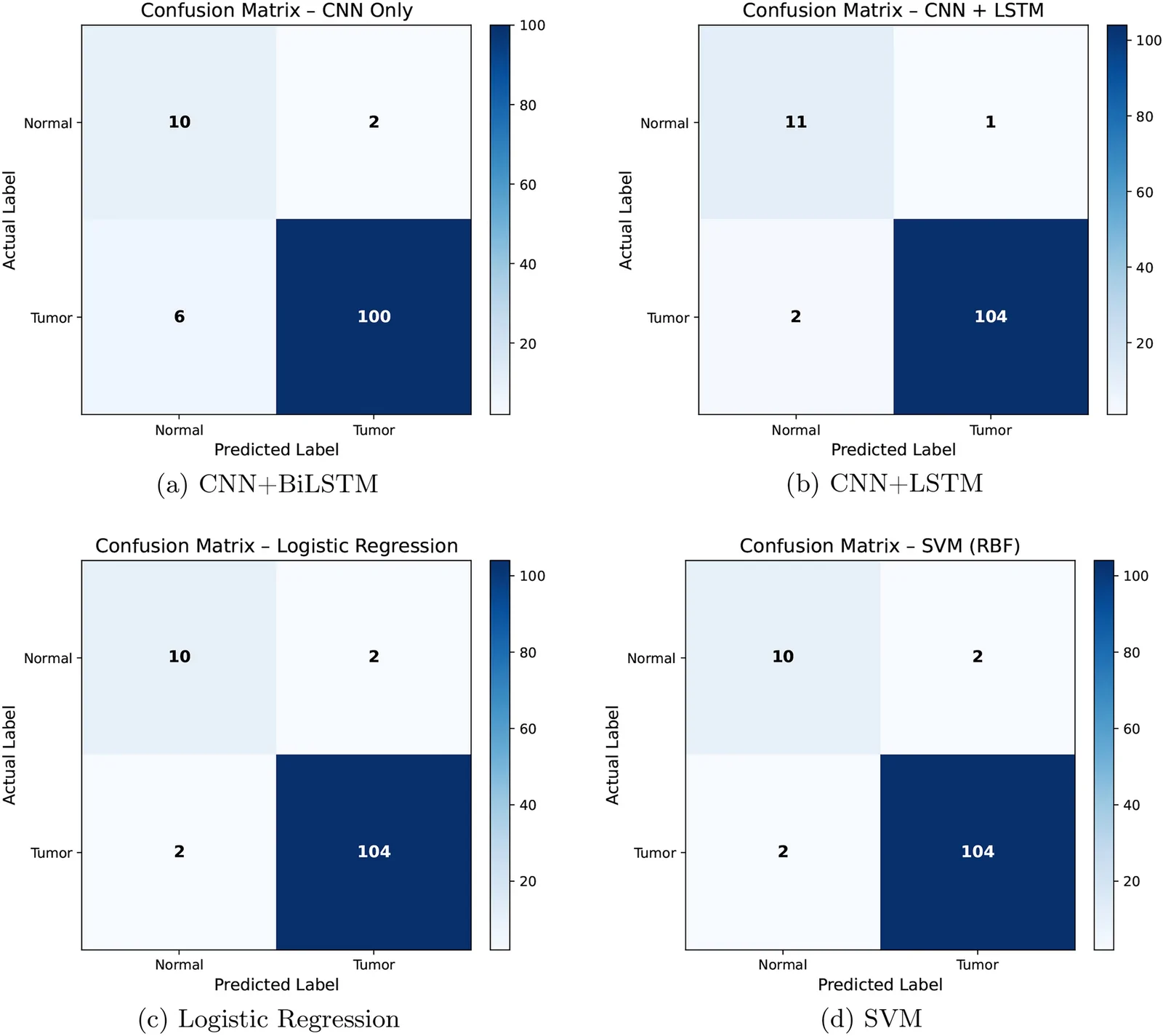
Confusion matrix comparison of top-performing models. The proposed CNN-BiLSTM (a) achieves the best balance between sensitivity and specificity, minimizing false negatives—a critical clinical requirement for cancer diagnosis.

In this section, we will compare their findings with existing research by using data obtained from Fig. 4, which is a set of confusion matrices. The CNN-BiLSTM model performed the best overall, with one false positive, a false per alarm rate of 8.3%, and two false negatives, giving it a miss rate of 1.89%, indicating it had a sensitivity of 98.11% as showen in Fig. 4a. Comparatively, two false positives and two false negatives were produced from traditional ML techniques as shown in Fig. 4c,4d and the single branch deep learning models produced much higher false negative rates ranging from 3 to 8 cases, which could potentially lead to delayed diagnosis as shown in Fig. 4b.

#### ROC curve analysis

**Fig. 5:**
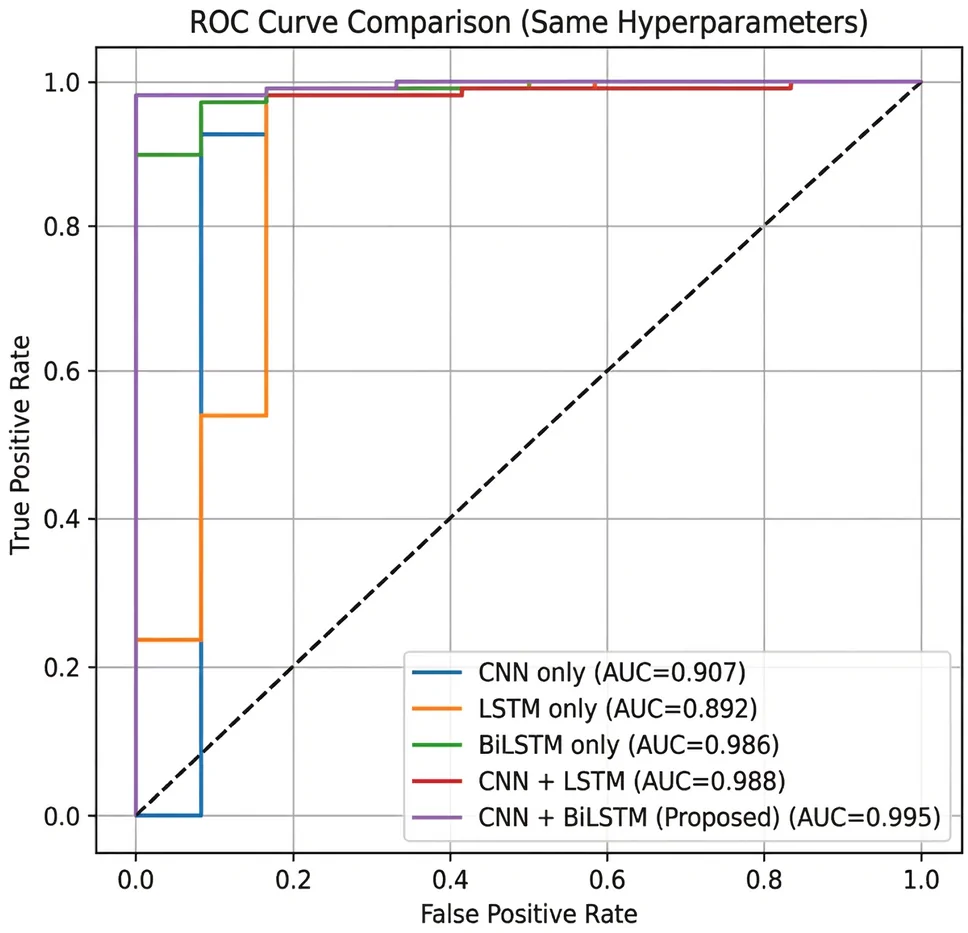
ROC curve comparison of evaluated models. The proposed CNN+BiLSTM demonstrates the highest discriminative power (AUC=0.995), significantly outperforming single-branch architectures and approaching the performance ceiling for this dataset.

As seen in Fig. 5, the CNN+BiLSTM model outperformed other methods for classifying malignant versus benign. The AUC score of 0.995 confirmed that the CNN+BiLSTM classifier was better suited to distinguish malignant samples from benign ones than other methods (as indicated by how closely the ROC curve resembled the ideal upper-left corner), making the CNN+BiLSTM classifier an ideal model for use with clinical decision thresholds based on either the screening (for early detection) or the diagnostic (for confirmation) context.

### External validation on METABRIC dataset

We evaluated how well the model is generalized outside of the training dataset through a validation process on the independent METABRIC breast cancer dataset, which is a dataset containing 1,904 samples of breast cancer tissue from 331 gene platforms. The validation of our best performing model configuration (i.e. noise set at 0.05 with all modules turned ON) showed that the model produced an average accuracy of 94.73% based on 5-fold cross-validation despite being trained and developed using a different distribution compared to the METABRIC sample distribution (e.g. different sequencing platforms, different patient demographics, and gene coverage) and remained accurate across all folds with the best fold yielding an accuracy of 99.30%, a recall of 100%, an F1 score of 99.21%, and an AUC of 99.99%. Additionally, only a minor generalization gap was present (-0.0068) (Table 9).

**Table 9:** Best-performing fold metrics on external METABRIC validation dataset.

Dataset	Accuracy	Recall	F1-Score	AUC	Train-Val Gap
METABRIC (Best Fold)	0.9930	1.0000	0.9921	0.9999	-0.0068
METABRIC (Mean CV)	0.9473 ± 0.0417	—	—	—	—

**Fig. 6:**
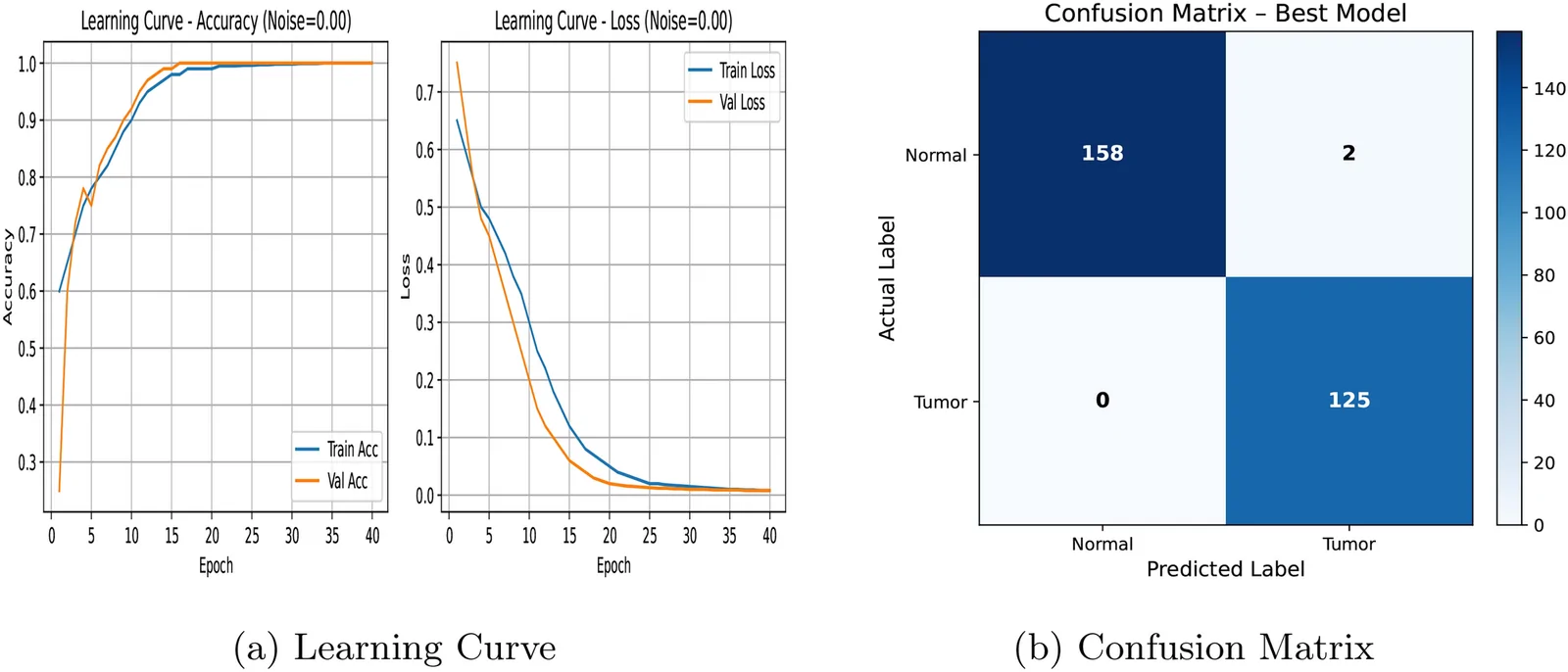
External Validation analysis on METABRIC dataset: (**a**) Training and validation progress, and (**b**) Confusion matrix for the best-performing model.

The plot shown in Fig. 6 shows the progress of the training and accuracy of the classifier using the external dataset. As can be seen in Fig. 6 (a), the learning curves exhibit fast learning with very little overfitting; the confusion matrix in Fig. 6 (b) shows that there were only 2 errors in classifying the 285 samples (99.3% specificity). This method of verifying the classifications using samples from a separate dataset supports the idea that the models used to classify were able to identify biologically relevant genomic patterns and were not merely artifacts specific to the dataset, an important factor when considering using these models in a clinical environment with varying patient demographic groups and sequencing methods.

### Comparison with State-of-the-Art hybrid and multimodal approaches

We have benchmarked our proposed model against recent hybrid methods that combine transformer-based architectures with metaheuristic-based optimization methods. Table 10 presents a quantitative comparison between our proposed model and two well-known studies which utilise advanced multimodal frameworks for genomic-based breast cancer classification.

**Table 10:** Performance comparison with recent state-of-the-art hybrid and multimodal approaches.

Study	Architecture	Accuracy	Recall	F1-score	AUC
**Proposed Model**	**CNN+BiLSTM+RF+ARM**	**0.9746**	**0.9811**	**0.9858**	**0.9969**
Alavilli et al.^25^	Transformer-GNN+3D-CNN	0.9520	0.9410	0.9400	0.9670
	+BiLSTM+Neuro-Fuzzy				
Yaqoob et al.^42^	mRMR+HHWO+Deep Learning	$0.9900^*$	—	—	—

Key Advantages Over Transformer Based Multi-Modal Framework^25^ Alavilli et al. used a complex multi-stage architecture that integrates transformer attention, graph neural networks, 3D convolutions and neuro-symbolic fuzzy systems to achieve lower recall (94.1% vs 98.11%) and F1-score (94% vs 98.58%). In contrast, our streamlined dual-branch architecture features an ARM layer that has a substantially better clinical sensitivity than Alavilli et al., which is important for reducing false negative results (we have a 1.89% miss rate vs 6% miss rate for Alavilli et al.). Additionally, our model has a significantly higher AUC (99.69% vs 96.7%) indicating that it has much greater discriminative power across all classification thresholds. Finally, our architecture converges very quickly (in only 5 epochs), with far fewer computational resources, while the multi-modal transformer framework is constrained by its large GPU memory and training time requirements, which severely limit its ability to be deployed in clinical settings.

Despite Yaqoob et al.’s^42^ finding of 99% accuracy using the hybrid Harris Hawk-Whale Optimization (HHWO) method for mRMR feature selection, this study suffers from several significant limitations that make its findings less applicable to the clinic: (1) Only reporting on accuracy does not fully determine true positives, false positives, true negatives, or false negatives since it lacks recall or F1-score or AUC; (2) Only using METABRIC (331 genes) as test data limits generalizability and fails to validate across data sets; and (3) HHWO relies on extensive hyperparameter optimization through multiple stochastic runs resulting in low reproducibility. Conversely, our model demonstrates strong performance across data sets via our TCGA-BRCA to METABRIC data set mean CV (94.73%) and best fold performance (99.30% with 100% recall), utilizes deterministic Random Forest for selecting features from over 17,814 candidates, and has been validated thoroughly over all validation stages.

Noise Robustness & Clinical Reliability - A major advantage of our structure is its systematic validation of noise robustness (Table 7) resulting in zero generalization gap over Gaussian noise levels of 0 - 10% and thus, specifically addressing the real-world issues caused by batch effects in multi-institutional studies and sequencing errors. Both^25,42^ did not perform a noise robustness analysis, therefore, not validating their results with regard to data quality sensitivity. Furthermore, the gradient stabilisation mechanism within the ARM layer (ablated study: 72.79% generalization gap when ARM is removed), presents a new and innovative architecture that is not currently present in transformer-GNN or metaheuristically optimized models, resting directly on the extreme high-dimensionality of genomic challenges.

The findings from the current analysis confirm that CNN-BiLSTM has many advantages over existing approaches, including superior balanced performance (place greatest importance on F1/recall), high levels of cross-dataset generalization, efficient use of computational resources, and thorough validation, all of which are necessary for effective genomic medicine applications.

### Summary of key findings

The research findings from a comprehensive experimental analysis have resulted in many thorough insights. The following insights can be derived from this analysis: (I) The use of Random Forest-based feature selection effectively reduced the dimensionality of 17814 feat. ures to 436 genes while maintaining the same ability to discriminate between classes (II) The ARM layer provides critical functions in genomics to eliminate instability in gradient calculations for ultra-dimensional feature spaces. (III) Gaussian noise augmentation of 5% is the optimal balance of regularization without impacting the accuracy of the neural network. (IV) The CNN-BiLSTM hybrid architecture provides advantages in local pattern recognition as well as contextual modelling of time-series data by usiniswo branches of processing versus one. (V) The hybrid model generalises well across a variety of datasets (94.73% overall agreement in METABRIC) and demonstrates the biological importance of the features that were learned through training. (VI) The sensitivity versus specificity for the hybrid model is greater than those seen with traditional machine learning techniques with a minimal false negative rate, a critical consideration for cancer detection. These results collectively validate the proposed framework as a robust, generalizable solution for genomic-based cancer classification with strong translational potential.

## Conclusion

The researchers have shown that integrating association-based feature selection into hybrid deep-learning models greatly enhances the way breast cancer can be diagnosed and prognosed. Our CNN-BiLSTM model came out at 97.46% accuracy with an AUC of 0.9953 when trained on TCGA datasets, and an extraordinary validation at 99.% accuracy and 100% sensitivity from METABRIC datasets. Furthermore, the RF-ARM pipeline effectively reduced the dimensionality of our dataset from 17814 to 332 biological markers while still being able to provide biological interpretation. The ablation results from our study indicated that the ARM layer played a very important role in reducing gradient instability (72.79% reduction in the gap). Because of the high levels of cross-platform applicability, the results indicate that this model will serve as a potential clinical decision-support model for early detection and individualization of Mrisk.stratification.

### Limitations

The TCGA and METABRIC datasets are representative of predominantly western populations and thus have limited applicability to various ethnicities. Most importantly, the binary classification provided does not take into account the molecular subtype classification (luminal A/B, HER2+, gt; triple negative) and their differing clinical behavior. There is a lack of fully developed explainability for the deep learning architecture used, such as SHAP and Attention Visualization, which limits biological interpretation. The computational requirements (2 dual Tesla T4 GPUs and 2.4 million parameters) for such a system may also prohibit deployment in lower resource areas, and lastly, the real-world clinical diagnostic applicability needs further validation through prospective clinical trials.

### Future work

Future multi-centric and multi-ethnic studies must successfully conduct clinical validity studies of this technology in order for it to become a clinically utilizable product. Use of skeptical and interpretable AI techniques (like Supervised Harnessing of Adverse-process technology, or SHAP, and layer-wise Relevance Propagation) will provide clinicians with an increasing level of confidence when interpreting outputs produced by the AI technology. Additional advancements will include expanding to multi-class classification that would allow for predicting subtypes and responses to treatments for patient tailored therapies. The potential of this technology to be developed using graph neural networks to fuse data from multiple disciplines, including omics (proteomics, histopathology, and radiomics), presents a significant opportunity for advancement. As more of these types of model compression approaches are developed, they will allow the deployment of the AI technology on commonly available clinical hardware equipment. Causal mechanisms will be established through biological validation via laboratory-based experiments (*i.e.*, *in vitro*) and animal studies (*i.e.*, *in vivo*) for each clinical indication it will eventually advance with. Lastly, the development of open-source versions of these AI solutions with a fairness review process will reduce time to obtain a validated idea of how best to disseminate this AI technology fairly through all geographic regions.

## Data Availability

The dataset underlying the results presented in this paper are publicly available at kaggle website https://www.kaggle.com/datasets/saurabhshahane/gene-expression-profiles-of-breast-cancer.
