# Correction: Hybrid tuned deep learning model for breast cancer diagnosis using genetic data

**DOI:** 10.1038/s41598-026-70814-w

**Published:** 2026-09-10

**Authors:** Farah Hesham, Mohamed M. Abbassy, Mohammed Abdalla

**Affiliations:** 1 Information Technology Program, The Egyptian-Korean Faculty of Technological Industry and Energy, Beni-Suef Technological University, Beni-Suef, Egypt; 2 Faculty of Computers and Artificial Intelligence, Beni-Suef University, Beni-Suef, Egypt

Correction to: *Scientific Reports* https://doi.org/10.1038/s41598-026-41643-8, published online 21 March 2026

The original version of this Article contained an error in the name of Mohamed M. Abbassy, which was incorrectly given as Mohammed M. Abbassy.

The original Article has been corrected.

